# Oral cavity infection by the SARS-CoV-2: emphasizing the essence of masking and peptide therapeutics

**DOI:** 10.1186/s43042-022-00213-z

**Published:** 2022-01-10

**Authors:** Glory Omini Ibiang, Joseph Malachi, Mercy Omini Ibiang, Daniel Kenechi Chukwudi, Olanrewaju Ayodeji Durojaye

**Affiliations:** 1 Department of Biological Sciences, Coal City University, Emene, Enugu State Nigeria; 2 Department of Molecular and Cell Biology, School of Life Sciences, University of Science and Technology of China, Hefei, China; 3 Department of Chemical Sciences, Coal City University, Emene, Enugu State Nigeria; 4 Department of Biochemistry, University of Nigeria, Nsukka, Enugu State Nigeria

**Keywords:** SARS-CoV-2, COVID-19, Oral cavity, ACE2, TMPRSS2

## Abstract

The SARS-CoV-2 has infected many people globally with the ravaging COVID-19; a disease, which has become challenging for every aspect of modern healthcare. The saliva and oral mucosa are sites of high risk for increased viral loads, and aside from the usual epithelial functions like lining and protection, the oral mucosa is also specialized for crucial functions, such as secretion, mastication, sensory perception, and taste perception. The human ACE2 receptor has been extensively studied for its essential role in the regulation of blood pressure homeostasis. However, scRNA-Seq studies have revealed high expression levels of the protein in keratinized epithelial surfaces of the oral cavity. The SARS-CoV-2 have access to the host’s body by binding to the ACE2 receptor, leading to the cleavage and major conformational changes in the viral spike glycoprotein for the release of its nucleocapsid into the cellular cytoplasm. This proteolytic cleavage is carried out by the TMPRSS2 and cathepsin L. In this study, we harnessed the information from the binding interface of TMPRSS2 and PAI-1 (a protease inhibitor known to inhibit the TMPRSS2 and several other proteases) to design a potential therapeutic peptide for the inhibition of the TMPRSS2, while also emphasizing the need for preventive masking.

**Supplementary Information:**

The online version contains supplementary material available at 10.1186/s43042-022-00213-z.

## Background

The COVID-19 (coronavirus disease 2019) is caused by the severe acute respiratory syndrome coronavirus 2 (SARS-CoV-2) and the World Health Organization (WHO) has categorized the virus as an airborne virus that can be transmitted by symptomatic, pre-symptomatic and asymptomatic patients, via affinity and exposure to both infected aerosols and droplets [1]. Although the transmission of the SARS-CoV-2 can be a result of activities that involve the oral cavity, like breathing, speaking, singing, sneezing, and coughing, attention is now mostly directed at the nasal and lung region of the infection. Oral expressions, such as the loss of taste, oral wounds and dry mouth have been noticed in about half of the patients infected with COVID-19, but whether the SARS-CoV-2 can infect directly and multiply in tissues of the mouth, like the mucosa or the salivary glands, remains elusive [1]. This is important because, the oral tissues, being early infection sites, could play crucial roles in the transmission of the virus to the gastrointestinal tracts or the lungs through the saliva, as observed in other diseases associated with microbes, like the inflammatory bowel diseases and pneumonia [2].

## Main text

SARS-CoV-2 infects using the entry factors of its host, such as the TMPRSS (transmembrane serine protease) family members (TMPRSS2 and 4) and the ACE2 (niotensin I-Converting Enzyme 2). A clearer understanding of the type of cell that harbors these entry factors is therefore crucial for the determination of susceptibility to SARS-CoV-2 infection throughout the body [3]. TMPRSS2 is an androgen‐responsive serine protease that promotes SARS-CoV-2 activation and entry through the cleavage of the viral spike glycoprotein. Aside from the lungs, many other tissues, such as the digestive tract, kidney and the cardiac endothelium, express the TMPRSS2, which suggests that these tissues might be crucial targets for SARS-CoV-2 infection [4]. The conversion of angiotensin II to angiotensin-(1–7) is catalyzed by the ACE2 and the ACE2/angiotensin-(1–7)/MAS axis counteracts the renin-angiotensin system (RAS) side effect, which plays a crucial role in the maintenance of the pathophysiological and physiological balance of the body. In addition to the direct effects of SAR-CoV-2, the immune and inflammatory factors associated with the pathogenesis of COVID-19, the imbalance and downregulation between the ACE2/angiotensin-(1–7)/MAS and RAS after infection may also promote multiple organ injury in COVID-19 [3].

The expression of TMPRSS2 and ACE2 has been documented in oral tissues but no detailed description of the direct confirmation of the SARS-CoV-2 infection nor the expression of viral entry factor in these tissues. Huang et al. [5] recently hypothesized that the barrier epithelia and salivary glands of the oropharynx and the oral cavity might be vulnerable to infection by the SARS-CoV-2 and can likewise be involved in the transmission process of the virus. In an attempt to test this hypothesis, the authors created two scRNA-seq atlases from the human mouth for the prediction of cell-specific SARS-CoV-2 infection susceptibilities. By this, the expression of TMPRSS2 and ACE2 in the oral mucosa epithelia and the salivary glands was confirmed. They confirmed SARS-CoV-2 infection using outpatient and autopsy specimens. Asymptomatic COVID-19 patients’ saliva also showed the likelihood of the transmission of the virus.

Public health operations, such as social distancing and the universal use of masks, are aimed at reducing the transmission of aerosols and droplets. However, only a few studies have tried to directly measure the variation in the ejection of saliva droplets from COVID-19 patients by wearing the mask. Huang et al. [5] therefore tested the effectiveness of standard mask-wearing in the reduction of the spread of droplets in these patients. The outcome from this study demonstrated a decrease above ten folds in the detected expelled salivary droplets [5].

The ongoing quest for the discovery of therapies for the COVID-19 pandemic is focused on the design of medications or vaccines aimed at the treatment and prevention of the disease. A major approach is the development of novel antiviral agents that are directed at the viral replication machinery, or host factors that are essential for the replication of the virus [6]. Serine proteases, which function through the activation of the spike glycoprotein of the virus and also aid the spread, replication and virus-cell membrane fusion for entry into the host cell, have been suggested as potential therapeutic targets for the development of antiviral drugs. Several literatures already provided evidences that the TMPRSS2 is one of such potential targets [6].

TMPRSS2 inhibitors can be divided into two groups, one of which includes different FDA-approved drugs. Examples of drugs in this group include Camostat, Aprotinin and Rimantadine [6]. The second group of TMPRSS2 inhibitors have been considered as potential therapeutic agents but are yet to be approved for human usage. Our focus in this study is on one of the group 2 potential inhibitors of the TMPRSS2 (the plasminogen activator inhibitor type 1). The plasminogen activator inhibitor type 1 (PAI-1) is an inhibitor of serine proteases, which regulates physiological blood clot breakdown through the inhibition of plasminogen activators in tissues, and urokinase [6]. However, the PAI-1 has also been reported as an effective TMPRSS2 inhibitor and likewise inhibits other serine proteases [3].

Dittmann et al. [7] in a previous study has reported the inhibitory potential of the PAI-1 against the TMPRSS2- and trypsin-mediated HA (surface glycoprotein hemagglutinin of influenza virus) cleavage, resulting in the suppression of the propagation of the H1N1 influenza virus both in vivo and ex vivo [7]. Here, we directed an in silico approach towards the modeling of the TMPRSS2-PAI-1 complex; a model, which to the best of our knowledge, is the first of its kind.

**Fig. 1:**
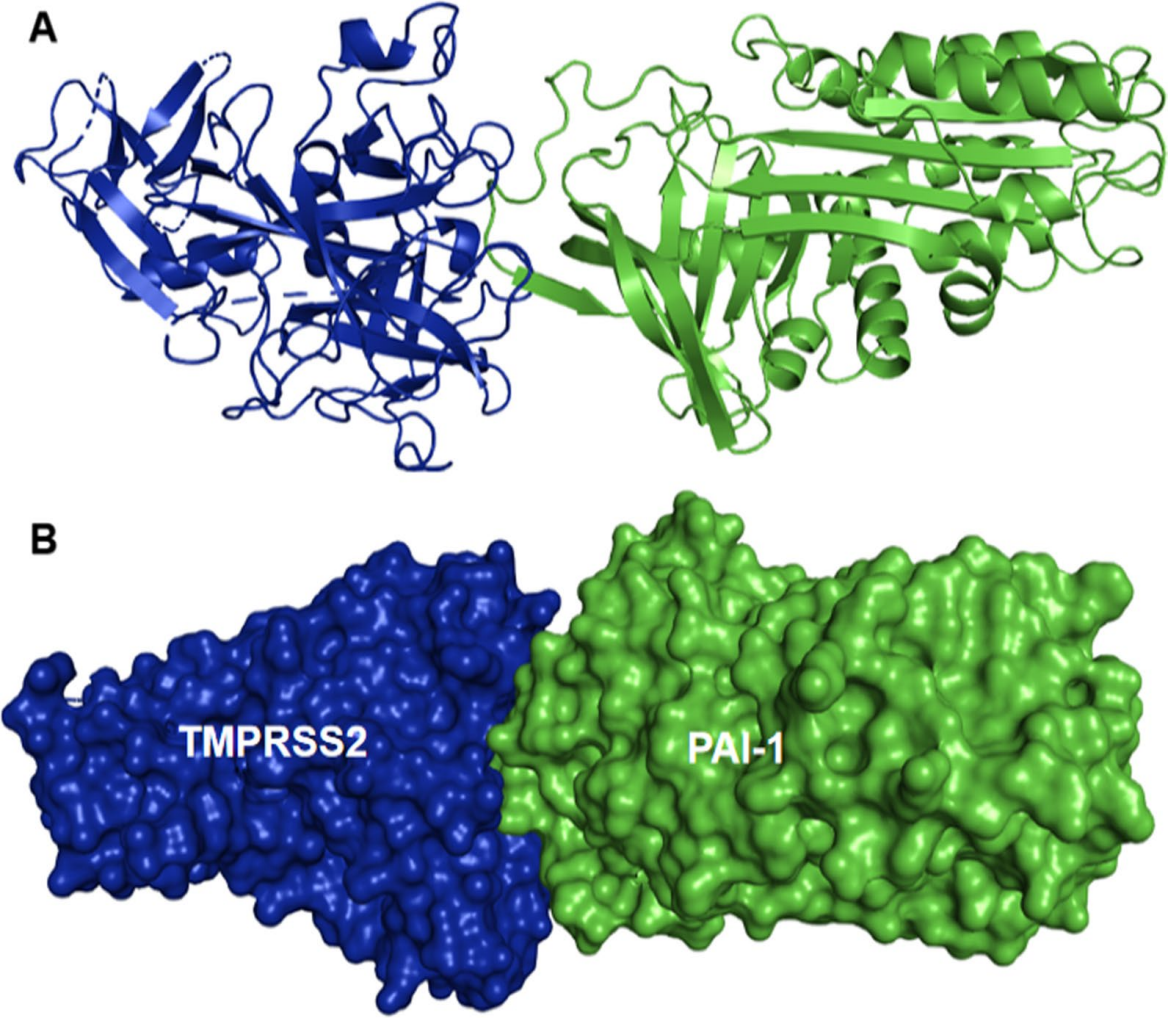
A 3D representation of the hypothetic TMPRSS2-PAI-1 complex. **a** Cartoon depiction of the predicted protein–protein interaction between TMPRSS2 (blue) and PAI-1 (green). **b** Surface depiction of the predicted protein–protein interaction between TMPRSS2 (blue) and PAI-1 (green)

The hypothetical model was generated through molecular docking with HDOCK [8]. The 3D structures of both interacting partners (TMPRSS2 and PAI-1) were obtained from the protein data bank with codes 7MEQ and 3CVM, respectively, while the final model of the docked complex was visualized using the PyMol molecular visualizer [9]. Furthermore, the binding free energy (MM/GBSA) of the protein complex upon docking was estimated using HawkDock [10] while the LigPlot software [11] was used for the visualization and analysis of interacting residues. The binding of PAI-1 to the catalytic site of TMPRSS2 suggests a stable complex (Fig. 1a, b), which is also evident from the HawkDock-deduced binding free energy calculation (− 182.38 kcal/mol) on per residue basis (Additional file 1: Table S1).

**Fig. 2:**
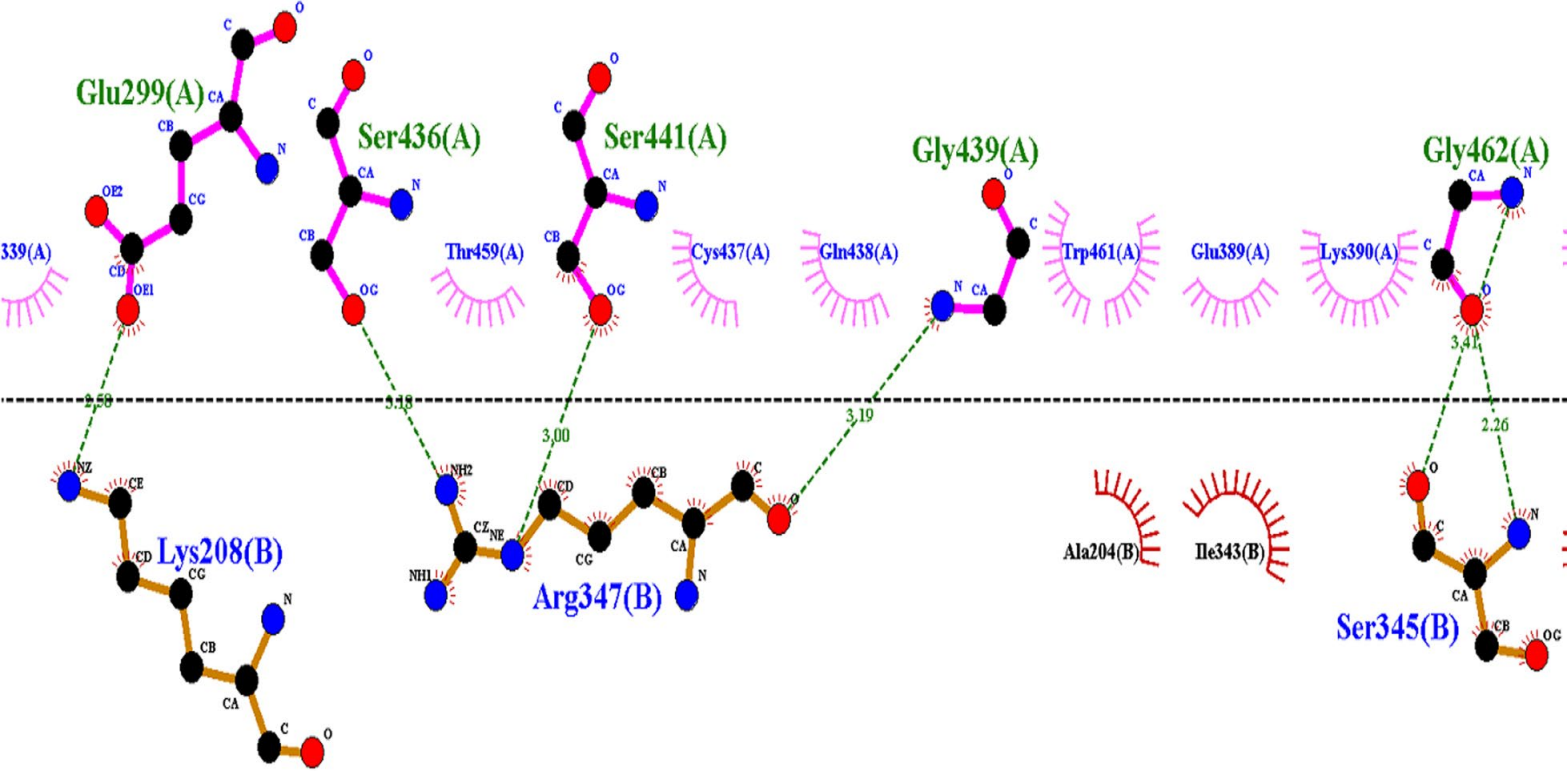
A 2D depiction of the predicted interaction between surface residues of the TMPRSS2-PAI-1 protein complex. Residues shown in the upper layer of the figure (colored green) denote the catalytic domain residues of TMPRSS2 while the lower layer residues denote the interacting surface residues of PAI-1. Each residue is named sequentially based on a combination of the 3-letter amino acid code, position, and chain

Proteins that possess a catalytic triad to use it either for the splitting of substrates (hydrolases) or for the transfer of a portion of one substrate to another (transferases). Catalytic triads are a set of interdependent residues in an enzyme active site and function in concert with other active site residues in order to achieve nucleophilic catalysis. Residues making up the catalytic triad act together towards making the nucleophile member highly reactive, thereby generating with the substrate a covalent intermediate which is then resolved for the completion of catalysis [12]. Several studies have identified the dependence of the function of TMPRSS2 proteolytic activation on the organization of a catalytic triad composed of the His-296, Asp-345 and Ser-441 residues [12]. Our analysis of the TMPRSS2-PAI-1 interaction predicts that the inhibitory role of PAI-1 is facilitated by the interaction of its Arg-347 residue with the catalytic triad residue of the TMPRSS2 (Ser-441) (Fig. 2), hence suggesting a destabilizing effect on the catalytic triad.

In spite of the therapeutic potentials of the PAI-1, the protein has been reported to be activated in several cancers, including oral cancer. Elevated PAI-1 expression in tumor tissues is considered as a prognostic marker of poor outcomes in the bulk of human cancer types, as the protein may be required for tumor growth and effective angiogenesis [13]. The increase in tumor growth as a result of the elevation of PAI-1 expression has been linked to the ability of the protein to effectively inhibit apoptosis [13]. In view of this drawback, we harnessed the information from the predicted TMPRSS2-PAI-1 binding interface for the design of a potential therapeutic peptide, to serve as an alternative for the inhibition of TMPRSS2.

**Fig. 3:**
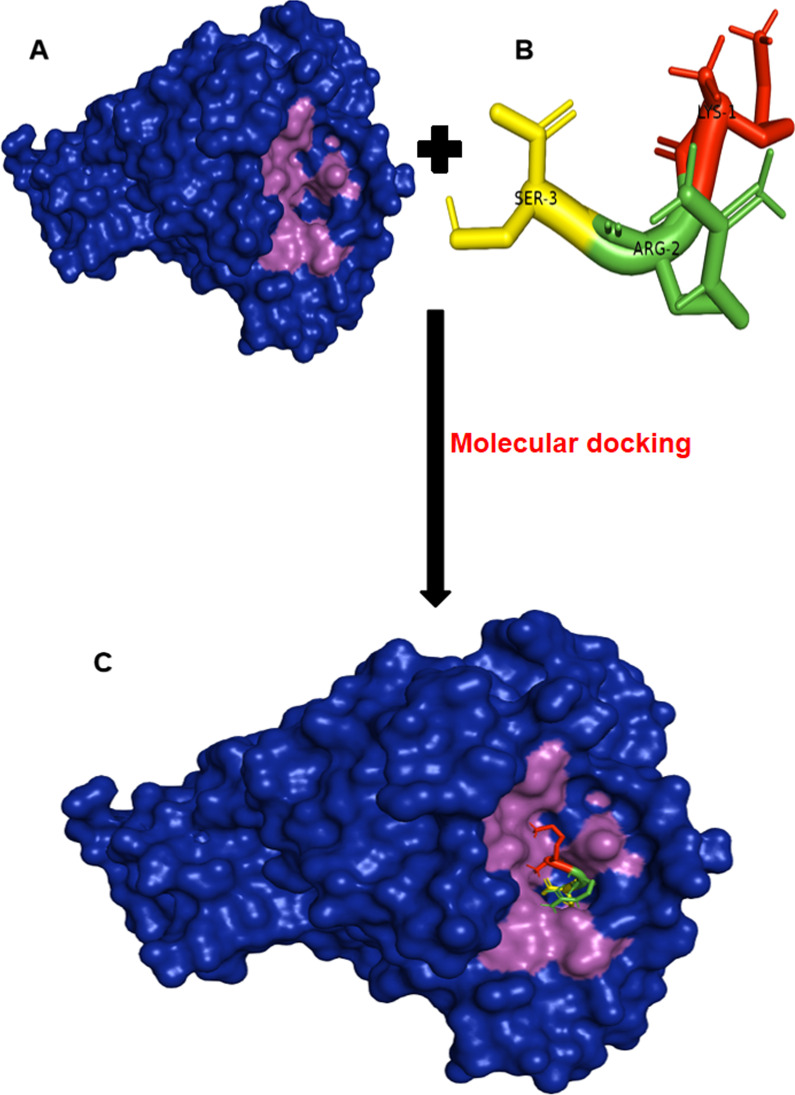
Peptide-based inhibition of the TMPRSS2 using the molecular docking protocol. **a** displays a surface representation of TMPRSS2 (blue) and the catalytic domain was distinguished using purple coloration. **b** Represent the designed therapeutic peptide with each residue shown in different colors and labeled according to the sequence of arrangement. Lys-1, Arg-2 and Ser-3 are all colored in red, green and yellow, respectively. **c** A display of the molecular docking result showing TMPRSS2 (surface) in a complex with the designed therapeutic peptide

As shown in Fig. 2, three residues on the PAI-1 binding interface (Lys-208, Arg-347, and SER-345) interacted with the catalytic domain residues of TMPRSS2. Following this sequence, we designed a potential therapeutic peptide (Fig. 3b) using the “build structure” function of the Chimera software [14]. The geometry of the generated 3D structure of the peptide was optimized prior to docking for the purpose of energy minimization, after which the structure was docked against the TMPRSS2 using the AutoDock Vina software [15]. Similarly, the binding free energy of the protein-peptide complex was estimated using the HawkDock tool, while interactions were analyzed using the LigPlot software [11].

**Fig. 4:**
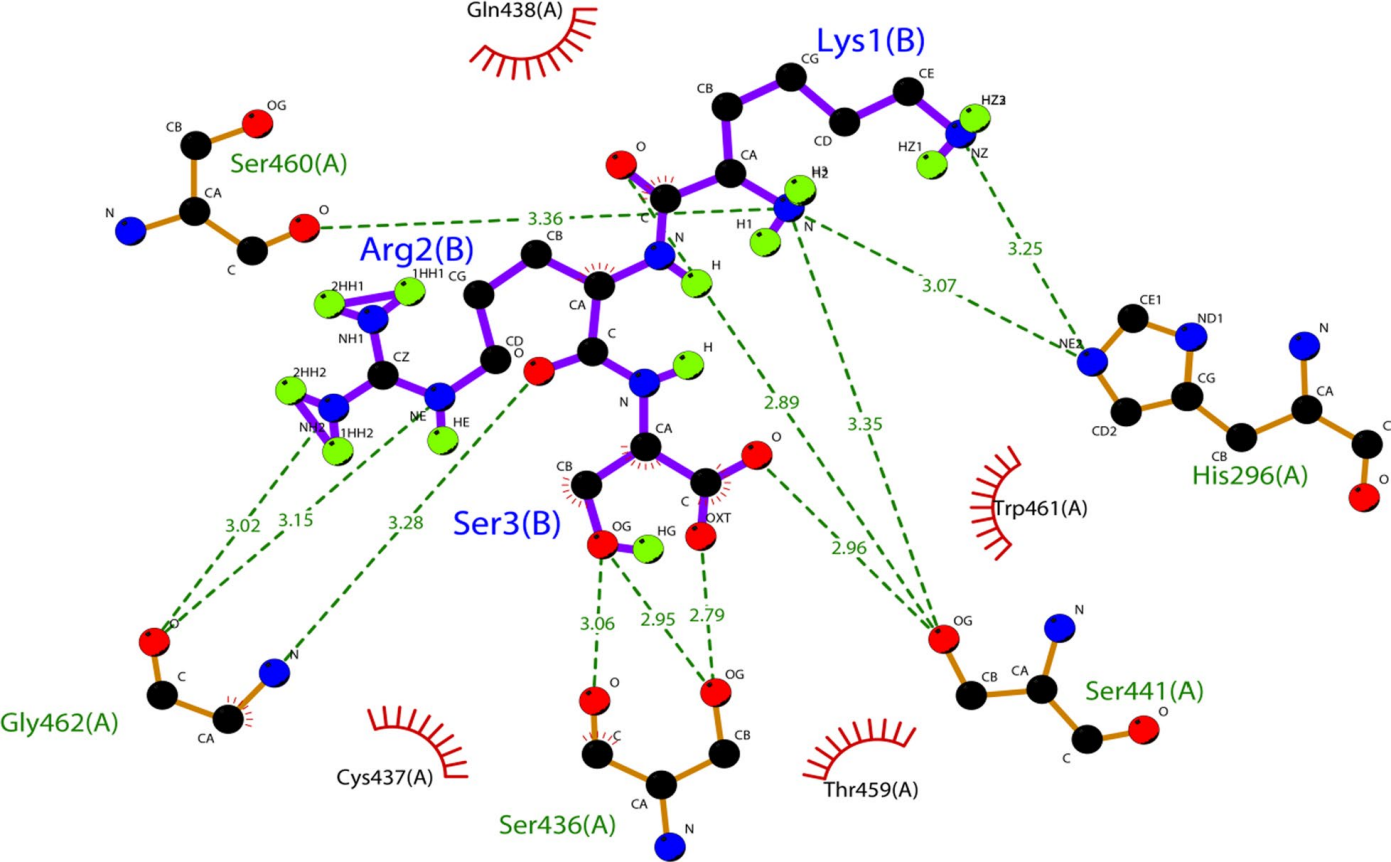
A 2D representation of the predicted interactions as a result of the therapeutic peptide binding to the catalytic domain of TMPRSS2 upon docking. Residues of the therapeutic peptide are labeled in blue color while their interacting partners (catalytic domain residues of TMPRSS2) are labeled in green color

Upon the docking of the therapeutic peptide against TMPRSS2 (Fig. 3), the generated binding free energy (− 266.25 kcal/mol) suggest that the peptide might exhibit a stronger affinity with the catalytic domain of TMPRSS2 (Additional file 1: Table S2) as compared to the generated binding free energy from the TMPRSS2-PAI-1 interaction (Additional file 1: Table S1). A careful study of the MM/GBSA output and interaction analysis (Fig. 4) also showed that the strong binding affinity of the therapeutic peptide can be linked to its interaction with important residues in the TMPRSS2 catalytic domain. The per-residue energy contribution analysis indicated that Ser-441, His-296 and Ser-460 are the highest energy contributors in the catalytic domain, with an energy contribution of − 21.89, − 14.05 and − 10.55 kcal/mol, respectively (Additional file 1: Table S2). This is also evident from the interaction analysis in Fig. 2 which shows that residues of the therapeutic peptide interact with the highest energy-contributing residues of the TMPRSS2 catalytic domain (Fig. 4).

Based on the nature of existing interactions between proteins, the development of low molecular weight compounds with the ability of reaching areas of the protein between 300 and 1000, have posed several challenges. This often results in a low binding affinity of the compounds. Medium-sized compounds (1000–2000 Da) are therefore suggested to be much more effective for the inhibition of protein–protein interactions [16]. Among the several groups of inhibitors with such properties, therapeutic peptides are the most widely studied. Peptides have several edges, ranging from the possibility of incorporating a variety of functional groups to the affordability of synthesis, and a direct similarity to protein fragments. Nevertheless, as a result of the low proteolytic stability of peptides, they have not preferred drug candidates. Short linear peptides have also been shown to exhibit low conformational stability, which might lead to a decrease in binding affinity to target proteins. However, various approaches have been applied in the development of peptide-based inhibitors, which could efficiently reduce the drawbacks [16].

Decreasing proteolytic cleavage susceptibility and increasing active conformation stability are the most crucial objectives for the introduction of peptide modifications. Two major types of structural changes are applied in peptide modifications; cyclizations and backbone modifications. The main effect of peptide cyclization is structural rigidification in the active state. Different strategies, such as hydrogen bond surrogates, hairpins, and stapling, have been built for the stabilization of extended conformations, turns, and helices [17]. The second method, which is based on the modification of backbone structures, usually alternates compound properties more completely, and the obtained 3D structures and sequences differ notably from the original protein fragment [17].

## Conclusion

In summary, the oral cavity represents an underappreciated and robust SARS-CoV-2 infection site, and despite the infection signs which include dry mouth, loss of taste, and mucosal injuries like macules, enanthema, and ulcerations, the direct role of the oral cavity in COVID-19 is yet to be fully understood. The exploration of its direct involvement in viral transmission, as elucidated in this study, therefore, necessitates the wearing of masks as a public health protective measure. Furthermore, protein–protein interaction targeting with the use of therapeutic peptides is a fast-growing pharmacological approach. A significant increase in the number of protein–protein interaction targets has been reported in recent times. However, many disease-related protein–protein interactions are yet to be discovered, considering the present knowledge of the human interactome. We have directed an extensive computational approach towards the 3D structure modeling of the TMPRSS2-PAI-1 complex, out of which a potential therapeutic peptide was designed through the exploration of the protein complex binding interface.

## Supplementary Information

**Additional file 1.** Per-residue free energy contribution table for the protein-protein interaction between TMPRSS2 and PAI-1.

**Additional file 2.** Per-residue free energy contribution table for the protein-peptide interaction between TMPRSS2 and the docked therapeutic peptide.

## Data Availability

Not applicable.

## Abbreviations

ACE2: Aniotensin I-Converting Enzyme 2
COVID-19: Coronavirus disease 2019
PAI-1: Plasminogen activator inhibitor type 1
RAS: Renin angiotensin system
scRNA-seq: Single-cell RNA sequencing
SARS-CoV-2: Severe acute respiratory syndrome coronavirus 2
TMPRSS2: Transmembrane serine protease 2
TMPRSS4: Transmembrane serine protease 4
