# Supplementary material for: Oral cavity infection by the SARS-CoV-2: emphasizing the essence of masking and peptide therapeutics
Source: Egypt J Med Hum Genet. 2022 Jan 10;23(1):1. doi: 10.1186/s43042-022-00213-z (PMC8744569; doi:10.1186/s43042-022-00213-z)
Supplement: Supplementary file 2 — Additional file 2. Per-residue free energy contribution table for the protein-peptide interaction between TMPRSS2 and the docked therapeutic peptide. [file 43042_2022_213_MOESM2_ESM.pdf]

Oral cavity 2

by Olayemi Hafeez RUFAI

Submission date: 09-Sep-2021 03:29AM (UTC-0400)
Submission ID: 1568362822
File name: Manuscript_review.docx (1.41M)
Word count: 3177
Character count: 18484

Oral cavity infection by the SARS-CoV-2: emphasizing the essence of masking and peptide therapeutics

Abstract

The SARS-CoV-2 has infected many people globally with the ravaging COVID-19; a disease which has become challenging for every aspect of modern healthcare. The saliva and oral mucosa are sites of high risk for increased viral loads, and aside the usual epithelial functions like lining and protection, the oral mucosa is also specialized for crucial functions, such as secretion, mastication, sensory perception, and taste perception. The human ACE2 receptor has been extensively studied for its essential role in the regulation of blood pressure homeostasis. However, scRNA-Seq studies have revealed high expression levels of the protein in keratinized epithelial surfaces of the oral cavity. The SARS-CoV-2 have access to the host's body by binding to the ACE2 receptor, leading to the cleavage and major conformational changes in the viral spike glycoprotein for the release of its nucleocapsid into the cellular cytoplasm. This proteolytic cleavage is carried out by the TMPRSS2 and cathepsin L. In this study, we harnessed the information from the binding interface of TMPRSS2 and PAI-1 (a protease inhibitor known to inhibit the TMPRSS2 and several other proteases) to design a potential therapeutic peptide for the inhibition of the TMPRSS2, while also emphasizing the need for preventive masking.

Keywords: SARS-CoV-2, COVID-19, Oral cavity, ACE2, TMPRSS2

To the Editor,

Background

The COVID-19 (coronavirus disease 2019) is caused by the severe acute respiratory syndrome coronavirus 2 (SARS-CoV-2) and the World Health Organization (WHO) has categorized the virus as an airborne virus that can be transmitted by symptomatic, pre-symptomatic and asymptomatic patients, via affinity and exposure to both infected aerosols and droplets [1]. Although the transmission of the SARS-CoV-2 can be as a result of activities that involves the oral cavity, like breathing, speaking, singing, sneezing and coughing, attention is now mostly directed at the nasal and lung region of the infection. Oral expressions, such as the loss of taste, oral wounds and dry mouth has been noticed in about half of the patients infected with COVID-19, but whether the SARS-CoV-2 can infect directly and multiply in tissues of the mouth, like the mucosa or the salivary glands remains elusive [1]. This is important because, the oral tissues, being early infection sites could play crucial roles in the transmission of the virus to the gastrointestinal tracts or the lungs through the saliva, as observed in other diseases associated with microbes, like the inflammatory bowel diseases and pneumonia [2].

Main text

SARS-CoV-2 infects using the entry factors of its host, such as the TMPRSS (transmembrane serine protease) family members (TMPRSS2 and 4) and the ACE2 (niotensin I-Converting Enzyme 2). A clearer understanding of the type of cell that harbors these entry factors is therefore crucial for the determination of susceptibility to SARS-CoV-2 infection throughout the body [3]. TMPRSS2 is an androgen-responsive serine protease that promotes SARS-CoV-2 activation and entry through the cleavage of the viral spike glycoprotein. Aside from the lungs, many other tissues, such as the digestive tract, kidney and the cardiac endothelium, expresses the TMPRSS2, which suggests that these tissues might be crucial targets for SARS-CoV-2 infection [4]. The conversion of angiotensin II to angiotensin-(1-7) is catalyzed by the ACE2 and the ACE2/angiotensin-(1-7)/MAS axis counteracts the renin-angiotensin system (RAS) side effect, which plays a crucial role in the maintenance of the pathophysiological and physiological balance of the body. In addition to the direct effects of SAR-CoV-2, the immune and inflammatory factors associated with the pathogenesis of COVID-19, the imbalance and downregulation between the ACE2/angiotensin-(1-7)/MAS and RAS after infection may also promote multiple organ injury in COVID-19 [3].

The expression of TMPRSS2 and ACE2 has been documented in oral tissues but no detailed description of the direct confirmation of the SARS-CoV-2 infection nor the expression of viral entry factor in these tissues. Huang *et al*. [5] recently hypothesized that the barrier epithelia and salivary glands of the oropharynx and the oral cavity might be vulnerable to infection by the SARS-CoV-2 and can likewise be involved in the transmission process of the virus. In an attempt to test this hypothesis, the authors

created two scRNA-seq atlases from the human mouth for the prediction of cell-specific SARS-CoV-2 infection susceptibilities. By this, the expression of TMPRSS2 and ACE2 in the oral mucosa epithelia and the salivary glands was confirmed. They confirmed SARS-CoV-2 infection using outpatient and autopsy specimen. Asymptomatic COVID-19 patients' saliva also showed the likelihood for the transmission of the virus.

Public health operations like social distancing and the universal use of masks, are aimed at reducing the transmission of aerosols and droplets. However, only a few studies have tried to directly measure the variation in the ejection of saliva droplets from COVID-19 patients by wearing mask. Huang *et al*. [5] therefore tested the effectiveness of standard mask wearing in the reduction of the spread of droplets in these patients. The outcome from this study demonstrated a decrease above ten folds in the detected expelled salivary droplets [5].

The ongoing quest for the discovery of therapies for the COVID-19 pandemic is focused on the design of medications or vaccines aimed at the treatment and prevention of the disease. A major approach is the development of novel antiviral agents that are directed at the viral replication machinery, or host factors that are essential for the replication of the virus [6]. Serine proteases, which function through the activation of the spike glycoprotein of the virus and also aid the spread, replication and virus-cell membrane fusion for entry into the host cell, have been suggested as potential therapeutic targets for the development of antiviral drugs. Several literature already provided evidences that the TMPRSS2 is one of such potential targets [6].

TMPRSS2 inhibitors can be divided into 2 groups, one of which include different FDA-approved drugs. Examples of drugs in this group include Camostat, Aprotinin and Rimantadine [6]. The second group of TMPRSS2 inhibitors have been considered as potential therapeutic agents but are yet to be approved for human usage. Our focus in this study is on one of the group 2 potential inhibitors of the TMPRSS2 (the plasminogen activator inhibitor type 1). The plasminogen activator inhibitor type 1 (PAI-1) is an inhibitor of serine proteases which regulates physiological blood clot breakdown through the inhibition of plasminogen activators in tissues, and urokinase [6]. However, the PAI-1 has also been reported as an effective TMPRSS2 inhibitor and likewise inhibits other serine proteases [7]

Dittmann *et al*. [8] in a previous study has reported the inhibitory potential of the PAI-1 against the TMPRSS2- and trypsin-mediated HA (surface glycoprotein hemagglutinin of influenza virus) cleavage, resulting in the suppression of the propagation of the H1N1 influenza virus both *in vivo* and *ex vivo* [8]. Here, we directed an *in silico* approach towards the modeling of the TMPRSS2-PAI-1 complex; a model which to the best of our knowledge is the first of its kind.

The hypothetical model was generated through molecular docking with HDOCK [9]. The 3D structures of both interacting partners (TMPRSS2 and PAI-1) were obtained from the protein data bank with codes 7MEQ and 3CVM respectively, while the final model of the docked complex was visualized using the PyMol molecular visualizer [10]. Furthermore, the binding free energy (MM/GBSA) of the protein complex upon docking was estimated using HawkDock [11] while the LigPlot software [12] was used for the visualization and analysis of interacting residues. The binding of PAI-1 to the catalytic site of TMPRSS2 suggest a stable complex (Fig. 1a and b), which is also evident from the HawkDock-deduced binding free energy calculation (-182.38 Kcal/mol) on per residue basis (Supplementary Table 1).

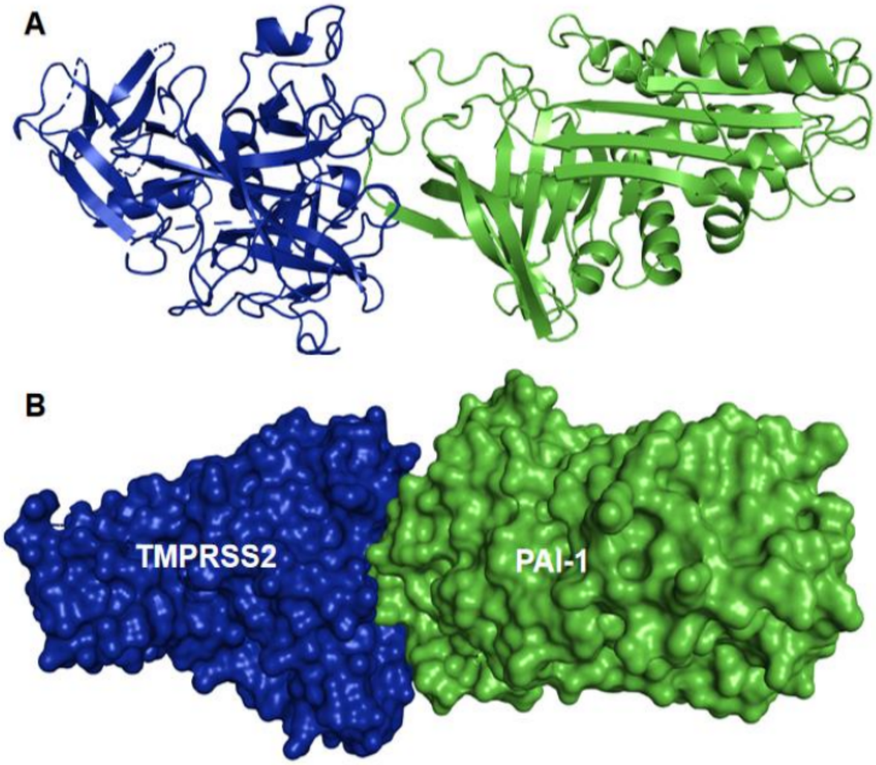

Figure 1. 3D representation of the hypothetical TMPRSS2-PAI-1 complex. (A) Cartoon depiction of the predicted protein-protein interaction between TMPRSS2 (blue) and PAI-1 (green). (B) Surface depiction of the predicted protein-protein interaction between TMPRSS2 (blue) and PAI-1 (green).

Proteins that possess a catalytic triad use it either for the splitting of substrates (hydrolases) or for the transfer of a portion of one substrate to another (transferases). Catalytic triads are a set of interdependent residues in an enzyme active site and function in concert with other active site residues in order to achieve nucleophilic catalysis. Residues making up the catalytic triad act together towards making the

nucleophile member highly reactive, thereby generating with the substrate a covalent intermediate which is then resolved for the completion of catalysis [13]. Several studies have identified the dependence of the function of TMPRSS2 proteolytic activation on the organization of a catalytic triad composed of the His-296, Asp-345 and Ser-441 residues [13]. Our analysis of the TMPRSS2-PAI-1 interaction predicts that the inhibitory role of PAI-1 is facilitated by the interaction of its Arg-347 residue with the catalytic triad residue of the TMPRSS2 (Ser-441) (Fig. 2), hence suggesting a destabilizing effect on the catalytic triad.

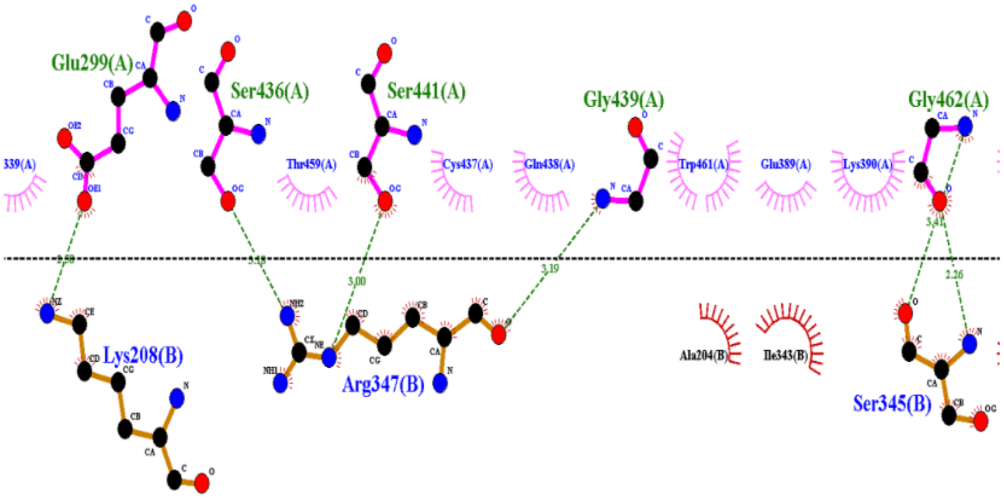

Figure 2. 2D depiction of the predicted interaction between surface residues of the TMPRSS2-PAI-1 protein complex. Residues shown in the upper layer of the figure (colored green) denote the catalytic domain residues of TMPRSS2 while the lower layer residues denote the interacting surface residues of PAI-1. Each residue is named sequentially based on a combination of the 3-letter amino acid code, position, and chain.

In spite of the therapeutic potentials of the PAI-1, the protein has been reported to be activated in several cancers including oral cancer. Elevated PAI-1 expression in tumor tissues is considered as a prognostic marker of poor outcome in the bulk of human cancer types, as the protein may be required for tumor growth and effective angiogenesis [14]. The increase in tumor growth as a result of the elevation of PAI-1 expression has been linked to the ability of the protein to effectively inhibit apoptosis [14]. In view of this drawback, we harnessed the information from the predicted TMPRSS2-PAI-1 binding interface for the design of a potential therapeutic peptide, to serve as an alternative for the inhibition of TMPRSS2.

As depicted in Figure 2, three residues on the PAI-1 binding interface (Lys-208, Arg-347, and SER-345) interacted with the catalytic domain residues of TMPRSS2.

Following this sequence, we designed a potential therapeutic peptide (Fig 3b) using the "build structure" function of the Chimera software [15]. The geometry of the generated 3D structure of the the peptide was optimized prior to docking for the purpose of energy minimization, after which the structure was docked against the TMPRSS2 using the AutoDock Vina software [16]. Similarly, the binding free energy of the protein-peptide complex was estimated using the HawkDock tool, while interactions were analyzed using the LigPlot software [12].

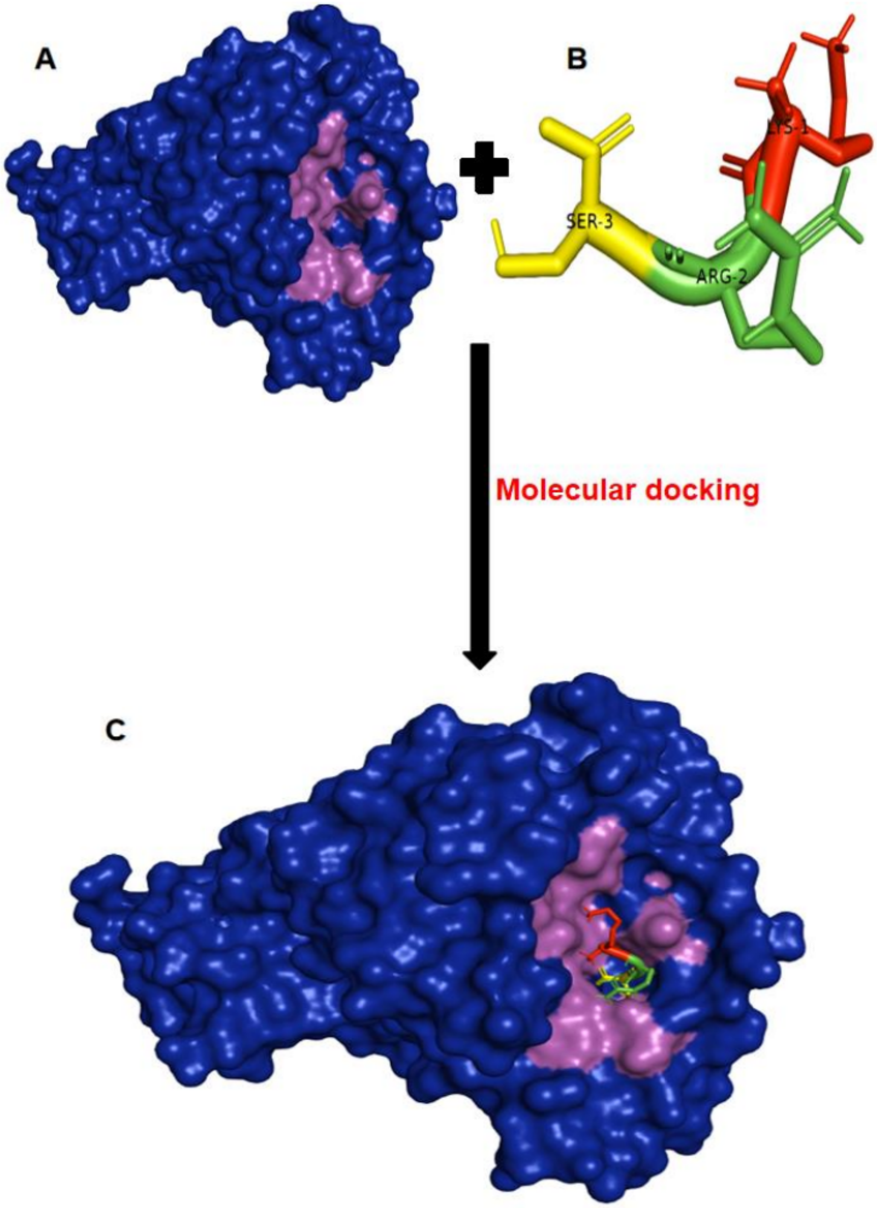

Figure 3. Peptide-based inhibition of the TMPRSS2 using the molecular docking protocol. (A) displays a surface representation of TMPRSS2 (blue) and the catalytic domain was distinguished using purple coloration. (B) represent the designed therapeutic peptide with each residue shown in different colors and labeled according to the sequence of arrangement. Lys-1, Arg-2 and Ser-3 all colored in red, green and yellow respectively. (C) a display of the molecular docking result showing TMPRSS2 (surface) in complex with the designed therapeutic peptide.

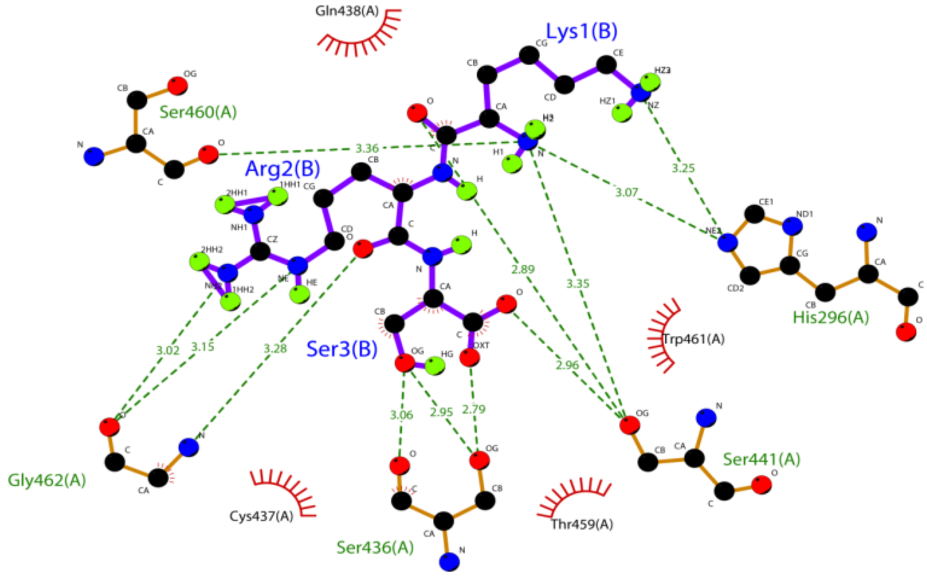

Figure 4. 2D representation of the predicted interactions as a result of the therapeutic peptide binding to the catalytic domain of TMPRSS2 upon docking. Residues of the therapeutic peptide are labeled in blue color while their interacting partners (catalytic domain residues of TMPRSS2) are labeled in green color.

Upon the docking of the therapeutic peptide against TMPRSS2 (Fig. 3), the generated binding free energy (-266.25 Kcal/mol) suggest that the peptide might exhibit a stronger affinity with the catalytic domain of TMPRSS2 (Supplementary Table 2) as compared to the generated binding free energy from the TMPRSS2-PAI-1 interaction (Supplementary Table 1). A careful study of the MM/GBSA output and interaction analysis (Fig. 4) also showed that the strong binding affinity of the therapeutic peptide can be linked to its interaction with important residues in the TMPRSS2 catalytic domain. The per-residue energy contribution analysis indicated that Ser-441, His-296 and Ser-460 are the highest energy contributors in the catalytic domain, with an energy contribution of -21.89, -14.05 and -10.55 Kcal/mol respectively (Supplementary Table 2). This is also evident from the interaction analysis in Figure 2 which shows that residues of the therapeutic peptide interact with the highest energy-contributing residues of the TMPRSS2 catalytic domain (Fig. 4).

Based on the nature of existing interactions between proteins, the development of low molecular weight compounds with the ability of reaching areas of the protein between 300 to 1000, have posed several challenges. This often result in a low binding affinity of the compounds. Medium-sized compounds (1000-2000 Da) are therefore suggested to be much more effective for the inhibition of protein-protein interactions [17]. Among the several groups of inhibitors with such properties, therapeutic peptides are the most widely studied. Peptides have several edges, ranging from the possibility of incorporating a variety of functional groups, to the affordability of synthesis, and a direct similarity to protein fragments. Nevertheless, as a result of the low proteolytic stability of peptides, they are not preferred drug candidates. Short linear peptides have also been shown to exhibit low conformational stability, which might lead to a decrease in binding affinity to target proteins. However, various approaches have been applied in the development of peptide-based inhibitors, which could efficiently reduce the drawbacks [17].

Decreasing proteolytic cleavage susceptibility and increasing active conformation stability are the most crucial objectives for the introduction of peptide modifications. Two major types of structural changes are applied in peptide modifications; cyclizations and backbone modifications. The main effect of peptide cyclization is for structural rigidification in the active state. Different strategies, such as hydrogen bond surrogate, hairpins, and stapling, have been built for the stabilization of extended conformations, turns and helices [18]. The second method, which is based on the modification of backbone structures, usually alternates compound properties more completely, and the obtained 3D structures and sequences differ notably from the original protein fragment [18].

Conclusion

In summary, the oral cavity represent an underappreciated and robust SARS-CoV-2 infection site, and despite the infection signs which include dry mouth, loss of taste and mucosal injuries like macules, enanthema and ulcerations, the direct role of the oral cavity in COVID-19 is yet to be fully understood. The exploration of its direct involvement in viral transmission, as elucidated in this study therefore necessitates the wearing of masks as a public health protective measure. Furthermore, protein-protein interaction targeting with the use of therapeutic peptides is a fast growing pharmacological approach. A significant increase in the number of protein-protein interaction targets has been reported in recent times. However, many disease-related protein-protein interactions are yet to be discovered, considering the present knowledge of the human interactome. We have directed an extensive computational approach towards the 3D structure modeling of the TMPRSS2-PAI-1 complex, out of which a potential therapeutic peptide was designed through the exploration of the protein complex binding interface.

Abbreviations

ACE2: Aniotensin I-Converting Enzyme 2
COVID-19: Coronavirus Disease 2019
PAI-1: Plasminogen Activator Inhibitor type 1
RAS: Renin Angiotensin System
scRNA-seq: Single-cell RNA sequencing
SARS-CoV-2: Severe Acute Respiratory Syndrome Coronavirus 2
TMPRSS2: Transmembrane Serine Protease 2
TMPRSS4: Transmembrane Serine Protease 4

References

1. Asakawa J, Mohrenweiser HW. Characterization of two new electrophoretic variants of human triosephosphate isomerase: stability, kinetic, and immunological properties. Biochem Genet. 1982 Feb;20(1-2):59-76. doi: 10.1007/BF00484936. PMID: 7092803.
2. Byrd KM, Gulati AS. The "Gum-Gut" Axis in Inflammatory Bowel Diseases: A Hypothesis-Driven Review of Associations and Advances. Front Immunol. 2021 Feb 19;12:620124. doi: 10.3389/fimmu.2021.620124. PMID: 33679761; PMCID: PMC7933581.
3. Hoffmann M, Kleine-Weber H, Schroeder S, Krüger N, Herrler T, Erichsen S, Schiergens TS, Herrler G, Wu NH, Nitsche A, Müller MA, Drosten C, Pöhlmann S. SARS-CoV-2 Cell Entry Depends on ACE2 and TMPRSS2 and Is Blocked by a Clinically Proven Protease Inhibitor. Cell. 2020 Apr 16;181(2):271-280.e8. doi: 10.1016/j.cell.2020.02.052. Epub 2020 Mar 5. PMID: 32142651; PMCID: PMC7102627.
4. Zang R, Gomez Castro MF, McCune BT, Zeng Q, Rothlauf PW, Sonnek NM, Liu Z, Brulois KF, Wang X, Greenberg HB, Diamond MS, Ciorba MA, Whelan SPJ, Ding S. TMPRSS2 and TMPRSS4 promote SARS-CoV-2 infection of human small intestinal enterocytes. Sci Immunol. 2020 May 13;5(47):eabc3582. doi: 10.1126/sciimmunol.abc3582. PMID: 32404436; PMCID: PMC7285829.
5. Huang, N., Pérez, P., Kato, T. *et al*. SARS-CoV-2 infection of the oral cavity and saliva. *Nat Med* (2021). https://doi.org/10.1038/s41591-021-01296-8.
6. Jankun, J. (2020). COVID-19 pandemic; transmembrane protease serine 2 (TMPRSS2) inhibitors as potential drugs . *Translation: The University of Toledo Journal of Medical Sciences*, *7*, 1–5. https://doi.org/10.46570/utjms.vol7-2020-361
7. Hoffmann M, Kleine-Weber H, Schroeder S, Krüger N, Herrler T, Erichsen S, Schiergens TS, Herrler G, Wu NH, Nitsche A, Müller MA, Drosten C, Pöhlmann S. SARS-CoV-2 Cell Entry Depends on ACE2 and TMPRSS2 and Is Blocked by a Clinically Proven Protease Inhibitor. Cell. 2020 Apr 16;181(2):271-280.e8. doi: 10.1016/j.cell.2020.02.052. Epub 2020 Mar 5. PMID: 32142651; PMCID: PMC7102627.
8. Dittmann M, Hoffmann HH, Scull MA, Gilmore RH, Bell KL, Ciancanelli M, Wilson SJ, Crotta S, Yu Y, Flatley B, Xiao JW, Casanova JL, Wack A, Bieniasz PD, Rice CM. A serpin shapes the extracellular environment to prevent influenza A virus maturation. Cell. 2015 Feb 12;160(4):631-643. doi: 10.1016/j.cell.2015.01.040. PMID: 25679759; PMCID: PMC4328142.
9. Yan Y, Zhang D, Zhou P, Li B, Huang SY. HDOCK: a web server for protein-protein and protein-DNA/RNA docking based on a hybrid strategy.

Nucleic Acids Res. 2017 Jul 3;45(W1):W365-W373. doi: 10.1093/nar/gkx407. PMID: 28521030; PMCID: PMC5793843.
10. Schrödinger, L., & DeLano, W. (2020). *PyMOL*. Retrieved from http://www.pymol.org/pymol
11. Gaoqi Weng, Ercheng Wang, Zhe Wang, Hui Liu, Feng Zhu, Dan Li, Tingjun Hou, HawkDock: a web server to predict and analyze the protein–protein complex based on computational docking and MM/GBSA, *Nucleic Acids Research*, Volume 47, Issue W1, 02 July 2019, Pages W322–W330, https://doi.org/10.1093/nar/gkz397.
12. Andrew C. Wallace, Roman A. Laskowski, Janet M. Thornton, LIGPLOT: a program to generate schematic diagrams of protein-ligand interactions, *Protein Engineering, Design and Selection*, Volume 8, Issue 2, February 1995, Pages 127–134, https://doi.org/10.1093/protein/8.2.127.
13. Schmitt CA, Bergey CM, Jasinska AJ, Ramensky V, Burt F, Svardal H, et al. (2020) *ACE2* and *TMPRSS2* variation in savanna monkeys (*Chlorocebus* spp.): Potential risk for zoonotic/anthroponotic transmission of SARS-CoV-2 and a potential model for functional studies. PLoS ONE 15(6): e0235106. https://doi.org/10.1371/journal.pone.0235106
14. Wyganowska-Świątkowska M, Jankun J. Plasminogen activation system in oral cancer: Relevance in prognosis and therapy (Review). Int J Oncol. 2015 Jul;47(1):16-24. doi: 10.3892/ijo.2015.3021. Epub 2015 May 22. PMID: 26004216.
15. Pettersen EF, Goddard TD, Huang CC, Couch GS, Greenblatt DM, Meng EC, Ferrin TE. UCSF Chimera--a visualization system for exploratory research and analysis. J Comput Chem. 2004 Oct;25(13):1605-12. doi: 10.1002/jcc.20084. PMID: 15264254.
16. Trott O, Olson AJ. AutoDock Vina: improving the speed and accuracy of docking with a new scoring function, efficient optimization, and multithreading. J Comput Chem. 2010 Jan 30;31(2):455-61. doi: 10.1002/jcc.21334. PMID: 19499576; PMCID: PMC3041641.
17. Wójcik P, Berlicki Ł. Peptide-based inhibitors of protein-protein interactions. Bioorg Med Chem Lett. 2016 Feb 1;26(3):707-713. doi: 10.1016/j.bmcl.2015.12.084. Epub 2015 Dec 24. PMID: 26764190.
18. Kawamoto SA, Coleska A, Ran X, Yi H, Yang CY, Wang S. Design of triazole-stapled BCL9 α-helical peptides to target the β-catenin/B-cell CLL/lymphoma 9 (BCL9) protein-protein interaction. J Med Chem. 2012 Feb 9;55(3):1137-46. doi: 10.1021/jm201125d. Epub 2012 Jan 24. PMID: 22196480; PMCID: PMC3286869.

Oral cavity 2

ORIGINALITY REPORT

14%	8%	14%	2%
SIMILARITY INDEX	INTERNET SOURCES	PUBLICATIONS	STUDENT PAPERS

PRIMARY SOURCES

1	Ni Huang, Paola Pérez, Takafumi Kato, Yu Mikami et al. "SARS-CoV-2 infection of the oral cavity and saliva", Nature Medicine, 2021<br>Publication	1%
2	Mohamed Hamdy Elkarow, Amr Hamdy. "A Suggested Role of Human Growth Hormone in Control of the COVID-19 Pandemic", Frontiers in Endocrinology, 2020<br>Publication	1%
3	"Activation of Viruses by Host Proteases", Springer Science and Business Media LLC, 2018<br>Publication	1%
4	dvd.sagepub.com<br>Internet Source	1%
5	accp1.onlinelibrary.wiley.com<br>Internet Source	1%
6	jbiomedsci.biomedcentral.com<br>Internet Source	1%

7	Esmaeil Behmard, Bijan Soleymani, Ali Najafi, Ebrahim Barzegari. "Immunoinformatic design of a COVID-19 subunit vaccine using entire structural immunogenic epitopes of SARS-CoV-2", Research Square, 2020<br>Publication	1 %
8	scholarworks.iupui.edu<br>Internet Source	1 %
9	Julie Teresa Marchesan, Blake M. Warner, Kevin Matthew Byrd. "The "oral" history of COVID - 19: Primary infection, salivary transmission, and post - acute implications", Journal of Periodontology, 2021<br>Publication	1 %
10	Milroy, Lech-Gustav, Tom N. Grossmann, Sven Hennig, Luc Brunsveld, and Christian Ottmann. "Modulators of Protein–Protein Interactions", Chemical Reviews<br>Publication	<1 %
11	coek.info<br>Internet Source	<1 %
12	Wójcik, Paulina, and Łukasz Berlicki. "Peptide-based inhibitors of protein–protein interactions", Bioorganic & Medicinal Chemistry Letters, 2016.<br>Publication	<1 %

www.spandidos-publications.com

13	Internet Source	<1 %
14	Jun Mori, Gavin Y. Oudit, Gary D. Lopaschuk. "SARS-CoV-2 perturbs the renin-angiotensin system and energy metabolism", American Journal of Physiology-Endocrinology and Metabolism, 2020<br>Publication	<1 %
15	Bintou A. Ahidjo, Marcus Wing Choy Loe, Yan Ling Ng, Chee Keng Mok, Justin Jang Hann Chu. "Current Perspective of Antiviral Strategies against COVID-19", ACS Infectious Diseases, 2020<br>Publication	<1 %
16	ro.ecu.edu.au<br>Internet Source	<1 %
17	H. Mamitsuka. "Essential Latent Knowledge for Protein-Protein Interactions: Analysis by an Unsupervised Learning Approach", IEEE/ACM Transactions on Computational Biology and Bioinformatics, 2005<br>Publication	<1 %
18	Lorenzatti Hiles, Guadalupe, Amanda Bucheit, John R. Rubin, Alexandra Hayward, Angelica L. Cates, Kathleen C. Day, Layla El-Sawy, L. Priya Kunju, Stephanie Daignault, Cheryl T. Lee, Monica Liebert, Maha Hussain, and Mark L.	<1 %

Day. "ADAM15 Is Functionally Associated with the Metastatic Progression of Human Bladder Cancer", PLoS ONE, 2016.
Publication

19	Muhammad Roomi, Yaser Khan. "Potential Compounds for the Inhibition of TMPRSS2", American Chemical Society (ACS), 2020<br>Publication	<1 %
20	egonw.github.io<br>Internet Source	<1 %
21	publichealth.jmir.org<br>Internet Source	<1 %
22	www.biorxiv.org<br>Internet Source	<1 %
23	www.frontiersin.org<br>Internet Source	<1 %
24	www.ncbi.nlm.nih.gov<br>Internet Source	<1 %
25	Khan Sharun, Kuldeep Dhama, Abhijit M. Pawde, Christian Gortázar et al. "SARS-CoV-2 in animals: potential for unknown reservoir hosts and public health implications", Veterinary Quarterly, 2021<br>Publication	<1 %
26	Neal G. Ravindra, Mia Madel Alfajaro, Victor Gasque, Nicholas C. Huston et al. "Single-cell	<1 %

longitudinal analysis of SARS-CoV-2 infection in human airway epithelium identifies target cells, alterations in gene expression, and cell state changes", PLOS Biology, 2021
Publication

---

27 MARZENA WYGANOWSKA-ŚWIĄTKOWSKA, JERZY JANKUN. "Plasminogen activation system in oral cancer: Relevance in prognosis and therapy (Review)", International Journal of Oncology, 2015 — <1%
Publication

---

28 Mei Zhong, Bingpeng Lin, Janak L. Pathak, Hongbin Gao et al. "ACE2 and Furin Expressions in Oral Epithelial Cells Possibly Facilitate COVID-19 Infection via Respiratory and Fecal–Oral Routes", Frontiers in Medicine, 2020 — <1%
Publication

---

29 Paolo Verdecchia, Claudio Cavallini, Antonio Spanevello, Fabio Angeli. "The pivotal link between ACE2 deficiency and SARS-CoV-2 infection", European Journal of Internal Medicine, 2020 — <1%
Publication

---

30 Yalda Rahbar Saadat, Seyed Mahdi Hosseiniyan Khatibi, Sepideh Zununi Vahed, Mohammadreza Ardalan. "Host Serine Proteases: A Potential Targeted Therapy for — <1%

COVID-19 and Influenza", Frontiers in Molecular Biosciences, 2021

Publication

Exclude quotes	On	Exclude matches	< 3 words
Exclude bibliography	On		

Oral cavity 2

PAGE 1

Prep. You may be using the wrong preposition.

Article Error You may need to remove this article.

S/V This subject and verb may not agree. Proofread the sentence to make sure the subject agrees with the verb.

Run-on This sentence may be a run-on sentence. Proofread it to see if it contains too many independent clauses or contains independent clauses that have been combined without conjunctions or punctuation. Look at the "Writer's Handbook" for advice about correcting run-on sentences.

P/V You have used the passive voice in this sentence. Depending upon what you wish to emphasize in the sentence, you may want to revise it using the active voice.

Article Error You may need to use an article before this word.

PAGE 2

P/V You have used the passive voice in this sentence. Depending upon what you wish to emphasize in the sentence, you may want to revise it using the active voice.

S/V This subject and verb may not agree. Proofread the sentence to make sure the subject agrees with the verb.

Article Error You may need to remove this article.

Article Error You may need to remove this article.

Article Error You may need to remove this article.

P/V You have used the passive voice in this sentence. Depending upon what you wish to emphasize in the sentence, you may want to revise it using the active voice.

Missing "," You may need to place a comma after this word.

Article Error You may need to use an article before this word.

PAGE 3

Article Error You may need to use an article before this word. Consider using the article **a**.

Wrong Article You may have used the wrong article or pronoun. Proofread the sentence to make sure that the article or pronoun agrees with the word it describes.

Article Error You may need to use an article before this word.

Article Error You may need to use an article before this word.

Frag. This sentence may be a fragment or may have incorrect punctuation. Proofread the sentence to be sure that it has correct punctuation and that it has an independent clause with a complete subject and predicate.

Frag. This sentence may be a fragment or may have incorrect punctuation. Proofread the sentence to be sure that it has correct punctuation and that it has an independent clause with a complete subject and predicate.

Frag. This sentence may be a fragment or may have incorrect punctuation. Proofread the sentence to be sure that it has correct punctuation and that it has an independent clause with a complete subject and predicate.

Missing "," You may need to place a comma after this word.

Article Error You may need to remove this article.

Article Error You may need to remove this article.

P/V You have used the passive voice in this sentence. Depending upon what you wish to emphasize in the sentence, you may want to revise it using the active voice.

Frag. This sentence may be a fragment or may have incorrect punctuation. Proofread the sentence to be sure that it has correct punctuation and that it has an independent clause with a complete subject and predicate.

Missing "," You may need to place a comma after this word.

Article Error You may need to use an article before this word.

Article Error You may need to remove this article.

Dup. You have typed two **identical words** in a row. You may need to delete one of them.

PAGE 7

Article Error You may need to use an article before this word.

Article Error You may need to use an article before this word.

Confused You have used **their** in this sentence. You may need to use **they're** instead.

Frag. This sentence may be a fragment or may have incorrect punctuation. Proofread the sentence to be sure that it has correct punctuation and that it has an independent clause with a complete subject and predicate.

Frag. This sentence may be a fragment or may have incorrect punctuation. Proofread the sentence to be sure that it has correct punctuation and that it has an independent clause with a complete subject and predicate.

PAGE 8

Article Error You may need to remove this article.

Article Error You may need to remove this article.

Prep. You may be using the wrong preposition.

Article Error You may need to use an article before this word.

Article Error You may need to remove this article.

Article Error You may need to use an article before this word. Consider using the article **the**.

S/V This subject and verb may not agree. Proofread the sentence to make sure the subject agrees with the verb.

PAGE 9

PAGE 10
